# Trait determinants of impulsive behavior: a comprehensive analysis of 188 rats

**DOI:** 10.1038/s41598-018-35537-7

**Published:** 2018-12-05

**Authors:** Ana Rosa Soares, Madalena Esteves, Pedro Silva Moreira, Ana Margarida Cunha, Marco Rafael Guimarães, Miguel Murteira Carvalho, Catarina Raposo-Lima, Pedro Morgado, Ana Franky Carvalho, Bárbara Coimbra, António Melo, Ana João Rodrigues, António José Salgado, José Miguel Pêgo, João José Cerqueira, Patrício Costa, Nuno Sousa, Armando Almeida, Hugo Leite-Almeida

**Affiliations:** 1 Life and Health Sciences Research Institute (ICVS), School of Medicine, University of Minho, Braga, Portugal; 2 ICVS/3B’s - PT Government Associate Laboratory, Braga/Guimarães, Portugal; 3 Department of General Surgery, Hospital of Braga, Braga, Portugal; 4 Present Address: Kavli Institute for Systems Neuroscience and Centre for Neural Computation, Norwegian University of Science and Technology, Trondheim, Norway

## Abstract

Impulsivity is a naturally occurring behavior that, when accentuated, can be found in a variety of neuropsychiatric disorders. The expression of trait impulsivity has been shown to change with a variety of factors, such as age and sex, but the existing literature does not reflect widespread consensus regarding the influence of modulating effects. We designed the present study to investigate, in a cohort of significant size (188 rats), the impact of four specific parameters, namely sex, age, strain and phase of estrous cycle, using the variable delay-to-signal (VDS) task. This cohort included (i) control animals from previous experiments; (ii) animals specifically raised for this study; and (iii) animals previously used for breeding purposes. Aging was associated with a general decrease in action impulsivity and an increase in delay tolerance. Females generally performed more impulsive actions than males but no differences were observed regarding delay intolerance. In terms of estrous cycle, no differences in impulsive behavior were observed and regarding strain, Wistar Han animals were, in general, more impulsive than Sprague-Dawley. In addition to further confirming, in a substantial study cohort, the decrease in impulsivity with age, we have demonstrated that both the strain and sex influences modulate different aspects of impulsive behavior manifestations.

## Introduction

Impulsive behavior manifests in normal individuals and is characterized by poorly conceived and prematurely expressed actions and choices which often lead to undesirable results^1,2^. Attributes include impaired action restraint and/or cancelation (impulsive action), and decision-making in the absence of adequate deliberation (impulsive choice), which is characterized by a tendency to choose smaller but immediate gratification over larger but delayed ones^2–5^.

While considered an adaptive behavior, impulsivity has mostly been studied in the context of neuropsychiatric diseases such as addiction, compulsive eating and aggressive behavior^3,6^. Evidence does suggest, however, a significant relationship between trait impulsivity and the increased propensity for the development of maladaptive behaviors^7–13^ – see for review^14–16^.

Two factors known to affect the manifestation of trait impulsivity are age and sex. Both in human populations^17–21^ – see for review^22,23^ – and in rodent studies^24–31^ – see for review^32^ -, impulsive behavior has generally been shown to decline with age. Nonetheless, in some studies this trend was mediated by other factors such as strain^28^ or sex^31^, while others either did not observe this association between age and impulsivity^33^ or observed an inverse relationship – i.e. young adult rats presenting greater impulsivity than adolescent counterparts^34^. Regarding sex, its role in action^25,35^ and choice^29,31,36–39^ impulsivity is much less clearly defined, often revealing contradictory results.

**Figure 1:**
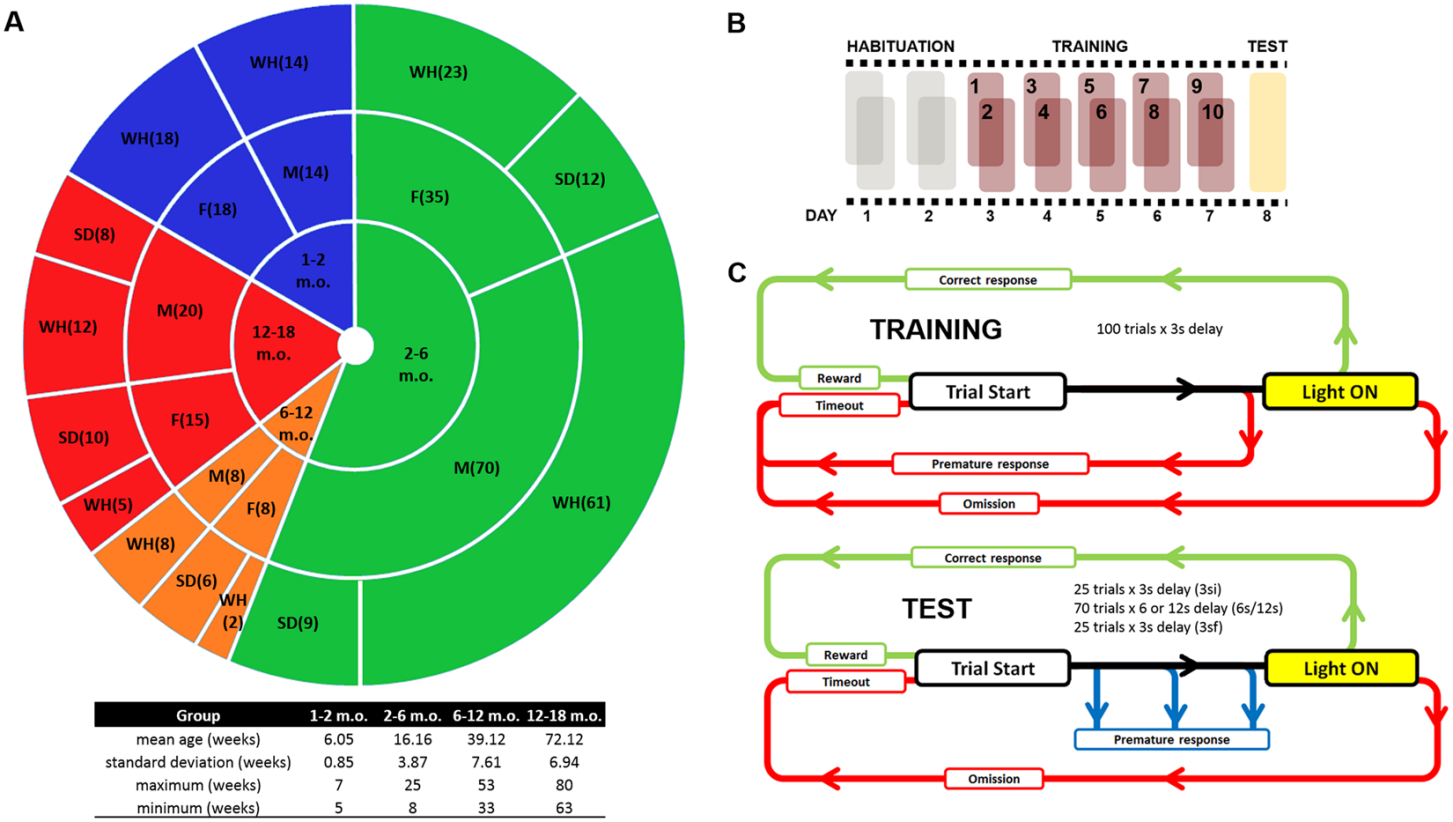
Experimental organization. (**A**) Composition of the groups assessed in this work (N = 188) by age, sex and strain. (**B**) The VDS protocol is comprised of 3 phases: habituation, training and test. Habituation and training are carried out in 4 and 10 sessions respectively, 2 sessions a day. The test session is performed in a single session on the last day. (**C**) Each training session (top) is comprised of 100 trials performed in a maximum of 30 minutes. During this phase, the animal learns to nosepoke following a light signal and correct responses are rewarded with a sugar pellet. If the animal does not respond to the light (omission) or responds before the light turns on (i.e., within the 3 s delay; premature response) no reward is delivered and the animal is punished with a 3 s timeout in complete darkness. The test session (bottom) is comprised of 120 trials performed in a maximum of 60 minutes. It is similar to training except that premature responses are allowed (i.e., not punished) and the delays vary according to the following schedule: 25 initial trials with 3 s delays (3si), 70 trials with randomized 6 s or 12 s delays (6s and 12s respectively) and 25 final trials with 3 s delays (3sf). F – female; M – male; WH – Wistar Han; SD – Sprague-Dawley.

In an attempt to further characterize the influence of biological parameters upon action and choice impulsivity, we collected and analyzed behavioral data from a large population of 188 rats, using the variable delay-to-signal (VDS) protocol (Fig. 1). The VDS is a previously validated paradigm which provides a rapid assessment of both impulsive action and delay tolerance (associated with choice impulsivity), and, as such, is particularly adequate for the characterization of large samples^40^.

## Results

### Task acquisition

**Figure 2:**
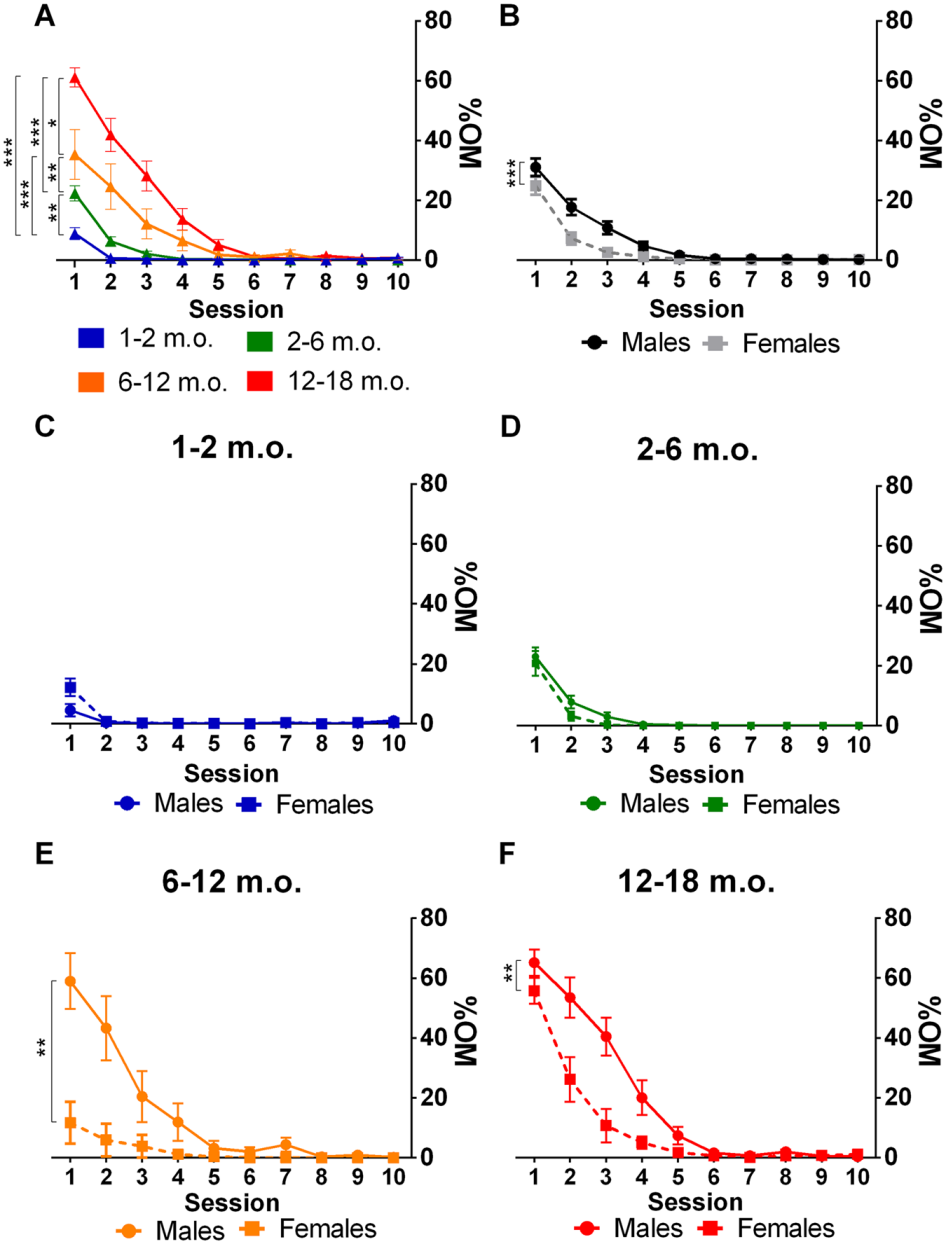
VDS task acquisition. Omissions as a function of session number by age and sex. (**A**) Older animals and (**B**) males require on average more sessions to reach a steady level of performance close-to-zero omissions. The effect of sex was particularly evident in (**E**) 6–12 and (**F**) 12–18 m.o. animals. Data is presented as mean ± SEM and statistically significant comparisons between groups are marked with *; *P < 0.05; **P < 0.01; ***P < 0.001; m.o. - months old; %OM - percentage of omissions.

The analysis of Mauchly’s test revealed that the assumption of sphericity was not met (χ^2^_(44)_ = 3515.1, p < 0.001). As a result, degrees of freedom (df) were corrected according to the Huynh-Feldt correction (ε = 0.236). There was a significant decrease in the percentage of omissions across the 10 training sessions (F_(2.2,309.8)_ = 109.40, p < 0.001, η_p_^2^ = 0.423). Significant between-subjects effects were observed for age (F_(3,149)_ = 31.58, p < 0.001, η_p_^2^ = 0.389), sex (F_(1,149)_ = 26.13, p < 0.001, η_p_^2^ = 0.149) and age*sex interactions (F_(3,149)_ = 8.73, p < 0.001, η_p_^2^ = 0.150). In addition, statistically significant age*session (F_(6.6,329.0)_ = 17.33, p < 0.001, η_p_^2^ = 0.259), sex*session (F_(2.2,329.0)_ = 12.12, p < 0.001, η_p_^2^ = 0.075) and age*sex*session (F_(6.6,329.0)_ = 5.53, p < 0.001, η_p_^2^ = 0.100) interactions were observed (Table 1 and Supplementary Table 1). The baseline number of omissions was found to increase with age. In addition, younger animals (1–2, 2–6 m.o.) effectively reached full performance (0 omissions) in 2–4 sessions while older animals (6–12 and 12–18 m.o.) took about 6 sessions to reach the same performance (Fig. 2A). Male animals displayed an increased mean number of omissions, an attribute which was particularly evident at the baseline (Fig. 2B). When age*sex*session effects were analyzed (Fig. 2C–F), it was observed that in older animals (6–12 and 12–18 m.o.), there was a significant difference between the sexes, with males presenting higher number of omissions in the first 4–5 sessions. Graphs, stratified by age and sex, depicting the full course of training can be found in Supplementary Fig. 1.

**Table 1:** General effects of sex and age on learning and action impulsivity.

	Training				Test
	%OM effects		%PR effects		PR rate 1^st^ sec effects
	Group	Group*Session	Group	Group*Session	Group
Age	F_(3,149)_ = 31.584; p < 0.001	F_(6.6,329.8)_ = 17.331; p < 0.001	F_(3,155)_ = 39.556; p < 0.001	F_(15.8,814.7)_ = 4.012; p < 0.001	F_(3,178)_ = 2.094; p = 0.103
Sex	F_(1,149__)_ = 26.132; p < 0.001	F_(2.2,329.8)_ = 12.118; p < 0.001	F_(1,155)_ = 33.726; p < 0.001	F_(5.3,814.7)_ = 4.918; p < 0.001	F_(1,178)_ = 10.554; p = 0.001
Sex*Age	F_(3,149)_ = 8.733; p < 0.001	F_(6.6,329.8)_ = 5.526; p < 0.001	F_(3,155)_ = 1.594; p = 0.193	F_(15.8,814.7)_ = 2.133; p = 0.006	F_(3,178)_ = 0.535; p = 0.659

Effects on task learning were assessed through the percentage of omissions during training (%OM) while effects on action impulsivity were evaluated in the percentage of premature responses during training (%PR) and prematurity rate during the first second of the test. Main effects of group (between factor) and session/group (within/between factors), in which groups are divided by age and sex, are shown. P < 0.05 was considered the threshold for statistical significance and age is measured in months.

### Impulsive action

**Figure 3:**
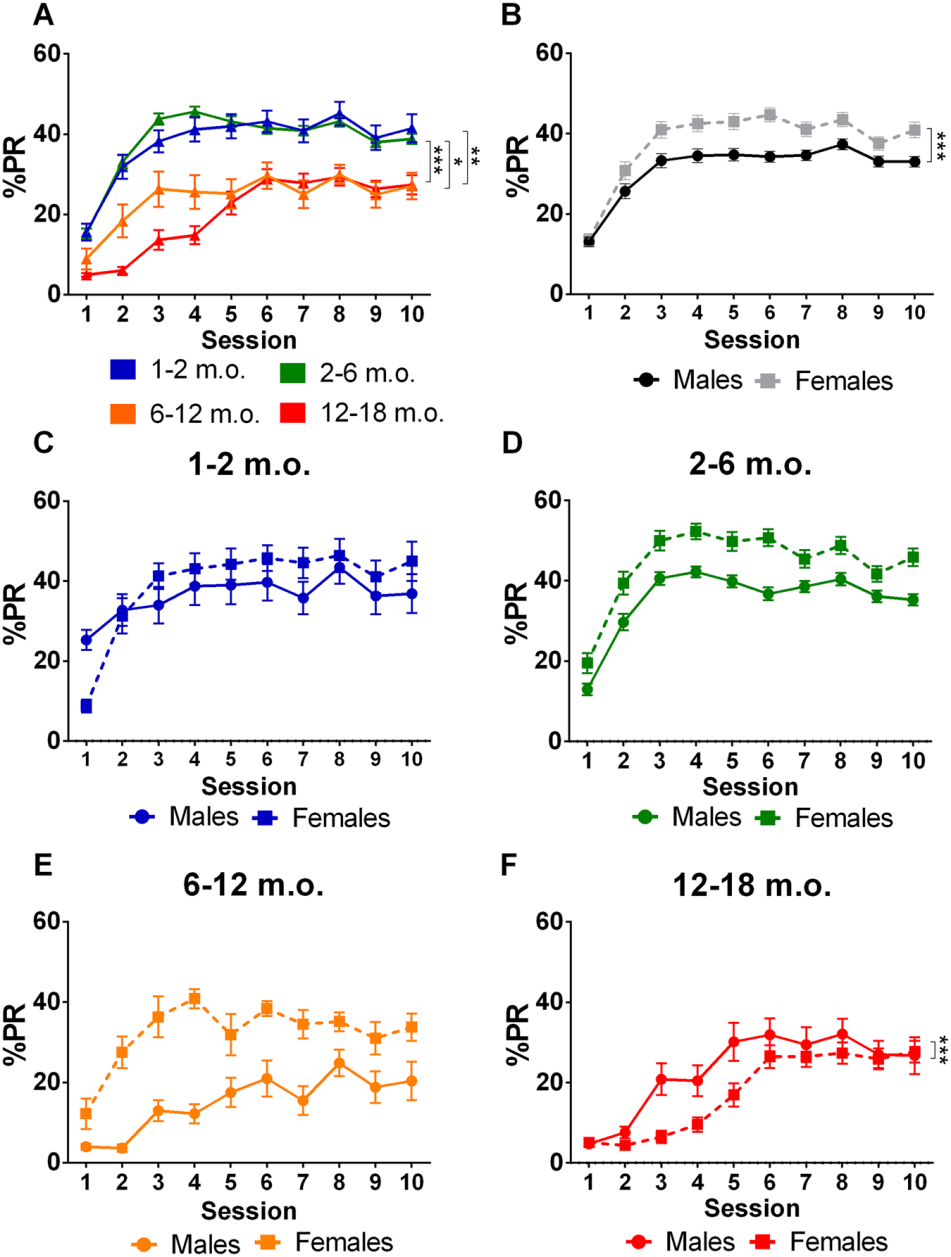
Action impulsivity (training phase). Percentage of premature responses as a function of session number by age and sex in the training phase. (**A**) Older animals and (**B**) males demonstrated less action impulsivity during training. Differences in behavior on the basis of sex for each age group was only evident in (**F**) 12–18 m.o. animals. Data is presented as mean ± SEM and statistically significant comparisons between groups are marked with *; *P < 0.05; **P < 0.01; ***P < 0.001; m.o. - months old; %PR - percentage of premature responses.

Similar to the percentage of omissions across training sessions, results from the mixed-design ANOVA violated the assumption of sphericity (χ^2^_(44)_ = 3312.8, p < 0.001) and consequently df were adjusted in accordance with a Huynh-Feldt correction (ε = 0.245). Significant increases (within-subjects effects) were observed for PR across training sessions (F_(5.3,814.7)_ = 70.72, p < 0.001, η_p_^2^ = 0.313). Between-subjects effects of age (F_(3,155)_ = 39.56, p < 0.001, η_p_^2^ = 0.434) and sex (F_(1,155)_ = 33.73, p < 0.001, η_p_^2^ = 0.179) were observed, but no significant interaction term between these variables (F_(3,155)_ = 1.59, p = 0.193, η_p_^2^ = 0.434) was evident. Post-hoc analysis revealed that the mean PRs across sessions were significantly higher for the younger animals (1–2 and 2–6 m.o.) in comparison to older animals (6–12 and 12–18 m.o.). Significantly more PRs were seen with females (M = 35.49%, SE = 1.24) than males (M = 25.26%, SE = 1.24). Analysis of within-between effects were significant for (i) age*session (F_(15.8,814.7)_ = 4.01, p < 0.001, η_p_^2^ = 0.072), (ii) sex*session (F_(5.3,814.7)_ = 4.92, p < 0.001, η_p_^2^ = 0.031), and (iii) age*sex*session interactions (F_(15.8,814.7)_ = 2.13, p = 0.006, η_p_^2^ = 0.040) (Table 1 and Supplementary Table 1). The assessment of the longitudinal trajectories for different groups indicated that (i) older animals displayed a constant, progressive increase in the number of PRs as session number increased, whereas younger animals displayed a disproportionate increase in the number of PRs during the first three sessions (Fig. 3A); (ii) both sexes displayed a similar number of PRs at baseline, but the rate of increase was greater in females until the third session (Fig. 3B); (iii) analysis of the age*sex*session interaction (Fig. 3C–F) revealed differences only in the 12–18 m.o. group, wherein males demonstrated a greater number of PRs (F_(4.2,96.5)_ = 5.36, P < 0.001; Fig. 3F) compared with females. This effect was not found when the analysis was restricted to sessions at which behavior was stabilized (i.e. sessions 6–10; F_(3.28, 85.33)_ = 1.23 p = 0.305), suggesting that the disparity may be attributed to learning (Fig. 2F), rather than to sex-related impulsivity differences.

**Figure 4:**
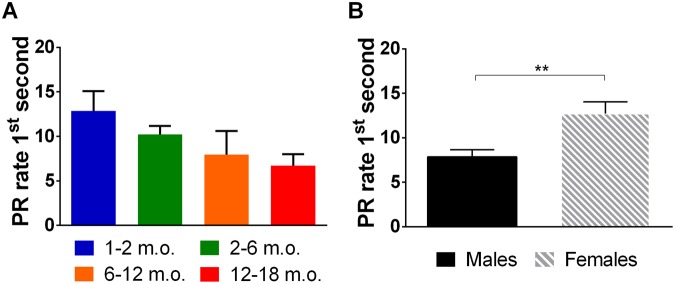
Action impulsivity (VDS). PR rate in the 1^st^ second of the 3si block of the test session by age and sex. **(A)** No age-dependent statistically significant differences were found. (**B**) males were found to demonstrate reduced action impulsivity in comparison with females. Sex differences for individual age groups are not plotted because no age vs sex interaction was found. Data are presented as mean ± SEM and statistically significant comparisons between groups are marked with *; **P < 0.01; m.o. - months old; PR rate - rate of premature responses per minute.

In the VDS test, first second PR rate in the 3si block constitutes an additional readout of action impulsivity (Fig. 4 and Table 1). Although, as in training, PR rate tended to decrease in an age-dependent manner (Fig. 4A), results from the univariate analysis of variance failed to achieve statistical significance (F_(3,178)_ = 2.09, p = 0.103, η_p_^2^ = 0.034). There were, however, statistically significant effects of sex on this parameter (F_(1,178)_ = 10.55, p = 0.001, η_p_^2^ = 0.056; Fig. 4B). No age*sex interaction was found in this measure (F_(__3,178__)_ = 0.54, p = 0.659, η_p_^2^ = 0.009).

### Delay intolerance

**Figure 5:**
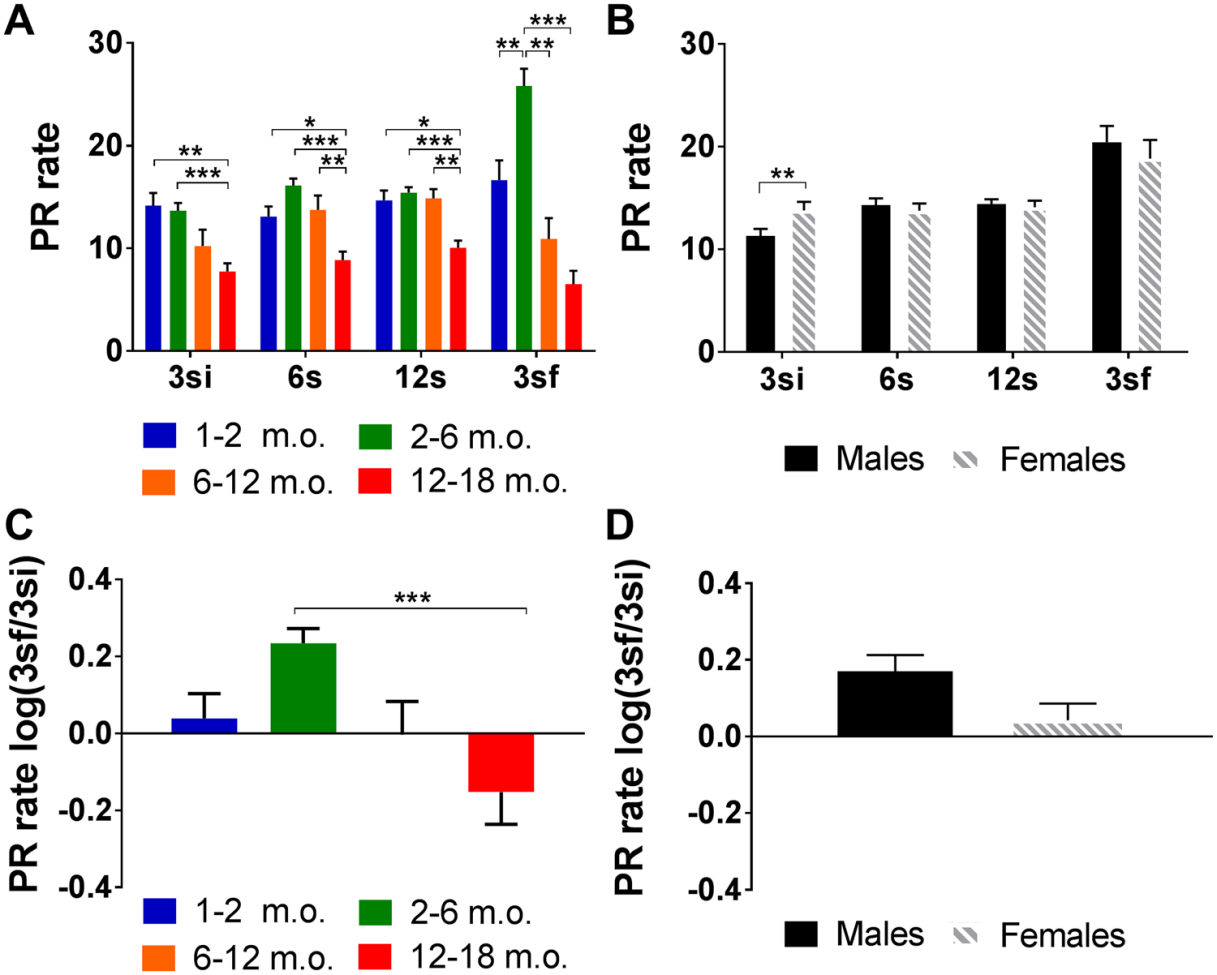
Delay tolerance. PR rate for each block of the test by age and sex. (**A**) The rate of premature responses decreased with age in all blocks, while (**B**) a sex difference was present only in the 3si interval. When responses in the final block (3sf) are compared to those in the initial block (3si), the same trend regarding both age (**C**) and sex (**D**) was found, i.e. there was a general decrease with age, but no effect of sex. Sex differences for individual age groups are not plotted because no age vs sex interaction was found in the 3sf block, or in the 3sf/3si. Data are presented as mean ± SEM and statistically significant comparisons between groups are marked with *; *P < 0.05; **P < 0.01; ***P < 0.001; m.o. - months old; PR rate - rate of premature responses per minute.

The PR rate decreased with age in all intervals (Table 2 and Supplementary Table 2) – 3si F_(3,176)_ = 8.30, p < 0.001 η_p_^2^ = 0.124; 6s F_(3,176)_ = 13.14, p < 0.001, η_p_^2^ = 0.183; 12s F_(3,176)_ = 9.60, p < 0.001, η_p_^2^ = 0.141; 3sf F_(3,176)_ = 18.92, p < 0.001, η_p_^2^ = 0.244 (Fig. 5A) – and the 3sf ratio to baseline 3si (F_(3,179)_ = 7.61, p < 0.001; Fig. 5C). Animals in the 12–18 m.o. group had the lowest PR rate in all intervals (Fig. 5A), and also demonstrated the greatest decrease in PR rate in the 3sf interval in comparison to baseline (log(3sf/3si); Fig. 5C). In contrast, the highest PR rate was generally seen in 2–6 m.o. animals (Fig. 5A), who also exhibited an increase in PR rate at 3sf (Fig. 5C).

**Table 2:** General effects of sex and age on delay intolerance.

	Test				
	PR rate effects				
	3si	6s	12s	3sf	log(3sf/3si)
Age	F_(3,176)_ = 8.298; p < 0.001	F_(3,176)_ = 13.138; p < 0.001	F_(3,176)_ = 9.602; p < 0.001	F_(3,176)_ = 18.919; p < 0.001	F_(3,179)_ = 7.607; p < 0.001
Sex	F_(1,176)_ = 10.845; p = 0.001	F_(1,176)_ = 0.987; p = 0.322	F_(1,176)_ = 0.051; p = 0.821	F_(1,176)_ = 0.400; p = 0.528	F_(1,179)_ = 0.499; p = 0.504
Sex*Age	F_(3,176)_ = 1.478; P = 0.222	F_(3,176_) = 2.518; p = 0.060	F_(3,176)_ = 2.481; p = 0.063	F_(3,176_) = 0.813; p = 0.488	F_(3,179)_ = 0.574; p = 0.632

Effects were assessed based on the prematurity rate (PR rate) during the test phases (3si, 6s, 12s and 3sf) and in the 3sf normalized to baseline (log(3sf/3si)). Main effects of group (age and sex) are shown. P < 0.05 was considered the threshold for statistical significance and age is measured in months.

Differences in performance related to sex (Table 2) were observed in the initial block, 3si (F_(1,176)_ = 10.85, p = 0.001, η_p_^2^ = 0.058; Fig. 5B) reflecting the same pattern observed in the training sessions, with females demonstrating higher PR rates. No other statistically significant sex differences or age vs sex interactions were observed in the intervals (Fig. 5B) or in the 3sf/3si comparison (Fig. 5D). See also Supplementary Fig. 2 for cumulative PRs throughout all intervals grouped by both age and sex.

### Response latency and latency to feed

**Figure 6:**
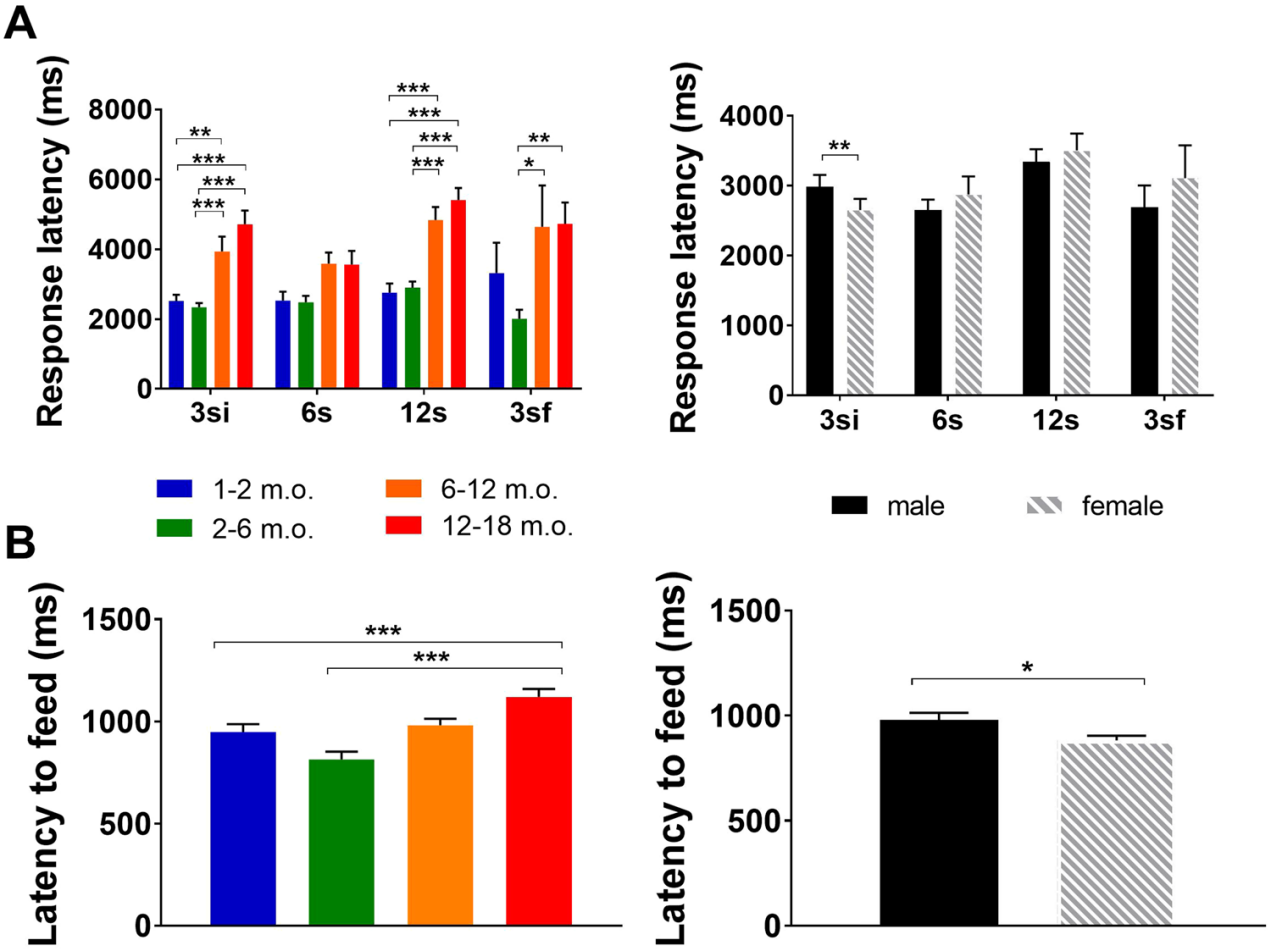
Response latency and latency to feed during the VDS test. (**A**) Response latency by age and sex. Response latency was influenced by age in all intervals except 6s, with older animals taking longer to perform a correct response (left). Males showed higher response latencies than females in the 3si, but no differences were found in the remaining intervals (right). (**B)** Overall latency to feed by age and sex. Latency to feed was influenced by age (left) and sex (right), with older (12–18 m.o.) and male animals showing increased time to retrieve the reward than younger (1–2 and 2–6 m.o. animals) and female animals, respectively. Data are presented as mean ± SEM and statistically significant comparisons between groups are marked with *; *P < 0.05; **P < 0.01; ***P < 0.001; m.o. - months old.

Latency to perform a (correct) response (Table 3) was affected by age in all intervals except 6 s (3si F_(3,180)_ = 15.13 p < 0.001; 6s F_(3,180)_ = 2.59 p = 0.054; 12s F_(3,180)_ = 16.75 p < 0.001; 3sf F_(3,180)_ = 4.44 p = 0.005), mostly reflecting higher latencies in older (6–12 and 12–18 m.o.) in comparison with younger (1–2 and 2–6 m.o.) animals (Fig. 6A left). Sex effects were only found in the 3si interval (F_(1,180)_ = 9.57; p = 0.002), where males showed higher latencies than females (Fig. 6A right). Age*sex interaction effects were found in all intervals, except 12s (3si F_(3,180)_ = 4.89 p = 0.003; 6s F_(3,180)_ = 3.34 p = 0.021; 12s F_(3,180)_ = 1.52 p = 0.212; 3sf F_(3,180)_ = 3.86 p = 0.010).

**Table 3:** General effects of sex and age on response latency.

	Test			
	Response latency effects			
	3si	6s	12s	3sf
Age	F_(3,180)_ = 15.131; p < 0.001	F_(3,180)_ = 2.589; p = 0.054	F_(3,180)_ = 16.754; p < 0.001	F_(3,180)_ = 4.441; p = 0.005
Sex	F_(1,180)_ = 9.566; p = 0.002	F_(1,180)_ = 1.261; p = 0.263	F_(1,180)_ = 0.003; p = 0.954	F_(1,180)_ = 0.798; p = 0.373
Sex*Age	F_(3,180)_ = 4.893; p = 0.003	F_(3,180)_ = 3.339; p = 0.021	F_(3,180)_ = 1.517; p = 0.212	F_(3,180)_ = 3.857; p = 0.010

Effects were assessed based on the response latency during the test phases (3si, 6s, 12s and 3sf). Main effects of group (age and sex) are shown. P < 0.05 was considered the threshold for statistical significance and age is measured in months.

Latency to feed (Table 4) was affected by age (F_(3,179)_ = 7.25, p < 0.001, η_p_^2^ = 0.108; Fig. 6B left), with older animals (12–18 m.o.) taking on average 244.6 and 143.9 ms more to recover the reward than 2–6 m.o. and 1–2 m.o. animals, respectively. Similarly, there was a significant association between sex and latency to feed (F_(1,179)_ = 4.00, p = 0.047, η_p_^2^ = 0.022; Fig. 6B right), whereas no age*sex interaction significant effects (F_(3,179)_ = 0.57, p = 0.636, η_p_^2^ = 0.009) were observed.

**Table 4:** Effects of sex and age on latency to feed.

	Test
	Group
Age	F_(3,179)_ = 7.249; p < 0.001
Sex	F_(1,179)_ = 3.997; p = 0.047
Sex*Age	F_(3,149)_ = 0.569; p = 0.636

Effects were assessed based on the latency to feed during the VDS test. Main effects of group (age and sex) are shown. P < 0.05 was considered the threshold for statistical significance and age is measured in months.

### Sub-sampling analyses

In order to explore the effect of strain and phase of estrous cycle upon impulsive behavior, additional analyses were performed. These were restricted to specific ages in order to obtain a homogenous group where variables were equally represented.

#### Strain

**Figure 7:**
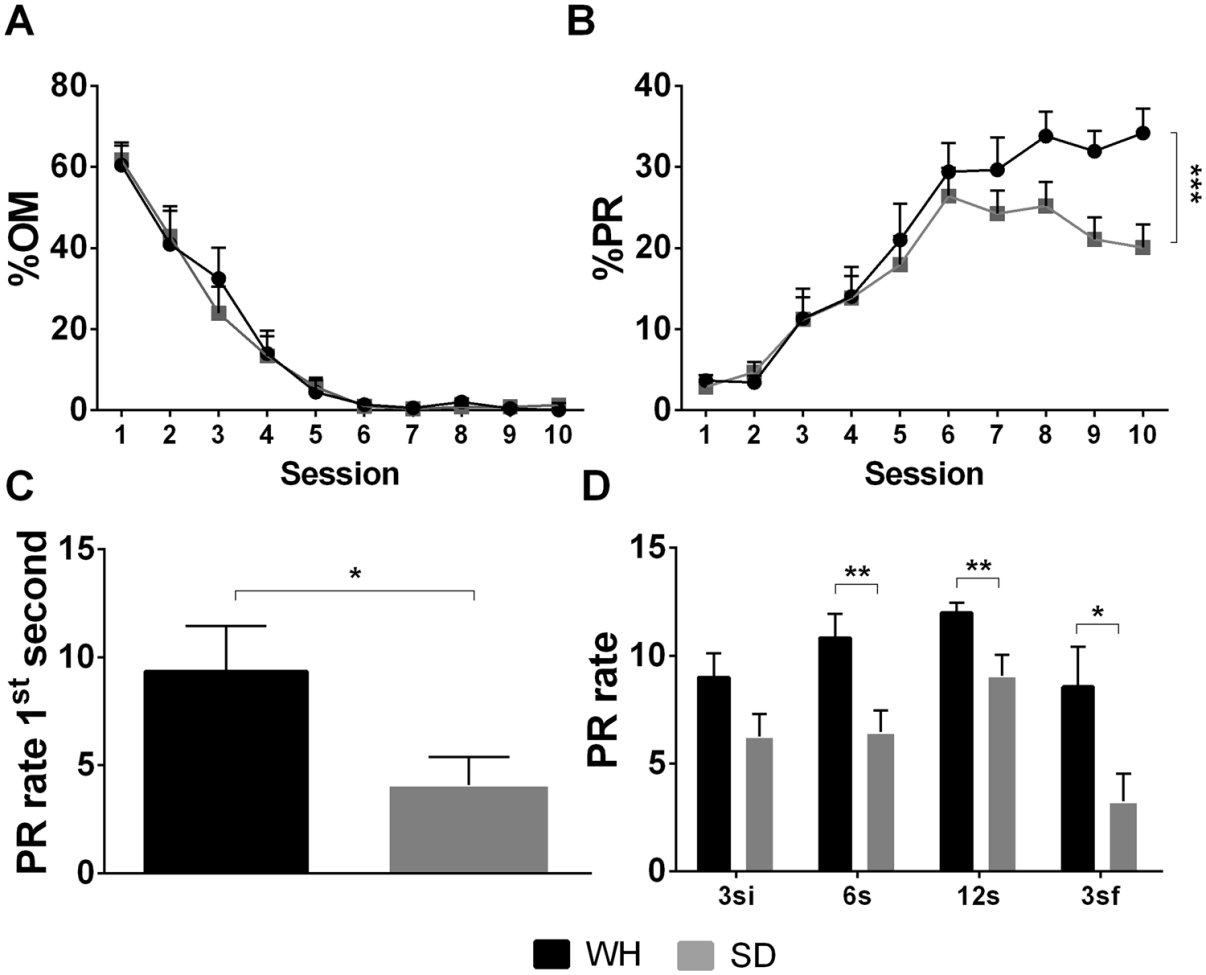
Influence of strain upon choice impulsivity and delay intolerance. The analysis of the effect of the strain in impulsive behavior was confined to 12–18 m.o. males and females. (**A**) Both strains learned the task equally well, progressively reducing %OM to 0. WH animals demonstrated higher action impulsivity in both (**B**) training and (**C**) PR rate in the 1^st^ second of 3si. (**D**) During the test, WH animals consistently showed higher PR rates in 6s, 12s and 3sf blocks. Data is presented as mean ± SEM and statistically significant comparisons between groups are marked with *; *P < 0.05; **P < 0.01; ***P < 0.001; %OM - percentage of omitted responses; %PR - percentage of premature responses; PR rate - rate of premature responses per minute; WH - Wistar Han; SD - Sprague-Dawley.

Analysis of the influence of strain, as well as strain*sex interaction on impulsive behavior was restricted to 12–18 m.o. males and females (SD: N = 18 (8 M, 10 F); WH: N = 17 (12 M, 5 F); Tables 5 and 6). Results from the mixed-design ANOVA (Huynh-Feldt corrected df: ε = 0.360), revealed no significant effect associated with either strain (F_(1,20)_ < 0.001, p = 0.984, η_p_^2^ = 0.148) or strain*session (F_(3.2,64.9)_ = 0.20, p = 0.910, η_p_^2^ < 0.001 upon the percentage of omissions (Fig. 7A). Regarding PR during training (Huynh-Feldt corrected df: ε = 0.820), no significant effect of strain was demonstrated (F_(1,21)_ = 3.63, p = 0.071, η_p_^2^ = 0.147), and no statistically significant strain*session interaction effects were observed (F_(7.4,154.9)_ = 5.60, p < 0.001, η_p_^2^ < 0.001; Fig. 7B). The 1^st^ second PR rate (F_(1,33)_ = 6.22, p = 0.018, η_p_^2^ = 0.172; Fig. 7C) did reveal that WH rats have higher rate of premature responses than SD animals. No significant effects of sex, or sex*strain interaction were seen in this group (see Table 5 section for detailed statistics).

**Table 5:** General effects of strain and sex on learning and action impulsivity.

	Training				Test
	%OM effects		%PR effects		PR rate 1^st^ sec effects
	Group	Group*Session	Group	Group*Session	Group
Strain	F_(1,20)_ < 0.001; p = 0.984	F_(3.2,64.9)_ = 0.197; p = 0.910	F_(1,21)_ = 3.625; p = 0.071	F_(7.4,154.92)_ = 5.596; p < 0.001	F_(1,33)_ = 6.224; p = 0.018
Sex	F_(1,20)_ = 5.066; p = 0.036	F_(3.2,64.9)_ = 4.295; p = 0.007	F_(1,21)_ = 11.556; p = 0.003	F_(7.4,154.92)_ = 4.760; p < 0.001	F(_1,33)_ = 3.011; p = 0.093
Strain*Sex	F_(1,20)_ = 2.488; p = 0.130	F_(3.2,64.9)_ = 1.518; p = 0.216	F_(1,21)_ = 2.111; p = 0.161	F_(7.4,154.92)_ = 1.089; p = 0.371	F_(1,33)_ = 0.072; p = 0.790

Effects on task learning were assessed through the percentage of omissions during training (%OM) while effects on action impulsivity were evaluated using the percentage of premature responses during training (%PR) and prematurity rate on the first second of the test. Main effects of group (between factor) and session/group (within/between factors), in which groups are divided by strain and sex, are shown. P < 0.05 was considered the threshold for statistical significance.

Examination of delay tolerance parameters revealed that WH animals present higher PR rate than SD counterparts in all intervals except 3si (Fig. 7D, Table 6) – 3si F_(1,33)_ = 3.52, P = 0.071, η_p_^2^ = 0.105; 6s F_(1,33)_ = 10.00, p = 0.004, η_p_^2^ = 0.250; 12s F_(1,33)_ = 8.45, p = 0.007, η_p_^2^ = 0.220; and 3sf F_(1,33)_ = 4.55, p = 0.042, η_p_^2^ = 0.231. The log(3sf/3si) analysis failed to demonstrate any differences between strains, or influence by either sex or sex*strain interaction.

**Table 6:** General effects of strain and sex on delay intolerance.

	Test				
	PR rate effects				
	3si	6s	12s	3sf	log(3sf/3si)
Strain	F_(1,33)_ = 3.515; p = 0.071	F_(1,33)_ = 10.002; p = 0.004	F_(1,33)_ = 8.446; p = 0.007	F_(1,33)_ = 4.552; p = 0.042	F_(1,33)_ = 0.484; p = 0.492
Sex	F_(1,33)_ = 1.095; p = 0.304	F_(1,33)_ = 0.126; p = 0.725	F_(1,33)_ = 0.788; p = 0.382	F_(1,33)_ = 0.353; p = 0.557	F_(1,33)_ = 1.321; p = 0.259
Strain*Sex	F_(1,33)_ = 0.418; p = 0.523	F_(1,33)_ = 4.992; p = 0.033	F_(1,33)_ = 7.881; p = 0.009	F_(1,33)_ = 0.474; p = 0.496	F_(1,33)_ = 0.081; p = 0.778

Effects were assessed based on the prematurity rate (PR rate) during the test phases (3si, 6s, 12s and 3sf) and in the 3sf normalized to baseline (log(3sf/3si)). Main effects of group (strain and sex) are shown. P < 0.05 was considered the threshold for statistical significance.

#### Estrous cycle

Females in the 2–6 m.o. age group were studied to assess the potential influence of phase of the estrous cycle (diestrus/proestrus) and impulsive behavior. Estrous cycle assessment was performed immediately after the VDS test (Diestrus: n = 9; Proestrus: n = 6) and only this phase was analyzed (Supplementary Fig. 3 and Supplementary Table 3). No differences were observed in the propensity for impulsive action as evaluated using the 1^st^ second of the 3si interval (F_(1,14)_ = 0.75, p = 0.402, η_p_^2^ = 0.055; Supplementary Fig. 3A). Estrous cycle phase had no influence upon behavior in any of the VDS blocks (Supplementary Fig. 3B) – 3si F_(1,12)_ = 0.93, p = 0.355, η_p_^2^ = 0.072; 6s F_(1,13)_ = 1.92, p = 0.189 η_p_^2^ = 0.129; 12s F_(1,12)_ = 3.50, p = 0.086, η_p_^2^ = 0.226; and 3sf F_(1,12)_ = 1.14, p = 0.307, η_p_^2^ = 0.087 - nor did it affect the variation to baseline PR rate (F_(1,14)_ < 0.01, *P* = 0.997; Supplementary Fig. 3 C). It is however noteworthy that, in all above-mentioned analyses, females in the proestrous phase showed a trend towards higher levels of impulsivity.

## Discussion

In the present study we characterized action impulsivity and delay tolerance in a group of 188 rats using the VDS test paradigm. The entire protocol lasts 8 days, but the VDS evaluation requires only a single session making it ideal to study short-lived phenomena or transient life stages such as adolescence or estrous cycle phases. An important attribute of the task is that it simultaneously captures both action impulsivity and delay tolerance. Task acquisition, reflected in the progressive decline of omission trials until complete fading, was achieved within 2–6 sessions. All animals were able to learn the task and from training sessions 7 to 10 virtually no omissions were recorded. Older animals typically required more sessions, an observation previously reported in other operant behavior protocols^41–44^.

Premature responses during the training phase reflect impulsive action. The behavioral construct is similar to that of the 5-choice serial reaction time task (5-csrtt)^45,46^. This type of impulsive behavior is also captured in the early moments of the 3si delays^40^. In both instances, impulsive action decreased with age. While a previous study in a 2-csrtt has shown that 1 m.o. rats were more impulsive than >3 m.o. animals^25^, the opposite was observed in a single instrumental nose poke task using animals of similar ages^34^. Interestingly, this last task bears some resemblances with the VDS test and results are probably more akin to delay intolerance rather than behavioral inhibition (see below). In a study confined to an older population, Muir and colleagues observed that 10–11 m.o. rats were more impulsive than 23–24 m.o. rats, though this observation was restricted to a specific condition (longer delays)^24^.

In the present study, sex was associated with distinct action impulsivity behavior, with females performing more premature responses than males. Similar findings have been reported in 3 m.o. rats using the 2-csrtt task, although the behavioral differences were manifested specifically in delays of longer duration^25^. The available literature indeed suggests that sex-related differences are specific of delay conditions – see for instance^25,35,47^.

The VDS test also includes a delay (in)tolerance component which correlates with delay discounting (DD) behavior and manifests as an increment of impulsive response rate upon exposure to large delays to signal/reward^40^ – see also^48–50^. In our population, 2–6 m.o. animals demonstrated increased PR rate in the 3sf block compared to baseline rate in 3si, while the remaining groups maintained (1–2 and 6–12 m.o.) or even slightly decreased (12–18 m.o.) their response rate. Our observations are consistent with DD protocols, which have demonstrated that 1 m.o. rats were less impulsive than 2 m.o. animals^34,51^ and that 25 m.o. rats were less impulsive than 6 m.o.^27^. Lukkes and colleagues also have observed that early adolescent female (but not male) rats were less impulsive in a DD task than were young adult/adult females^31^. It is difficult to construct a meaningful framework for the DD studies reported in the literature. On one hand, no age-related differences were demonstrated in one spatial adjusting-delay task (5, 9 and >27 m.o.)^33^, while in another study, 1 m.o. rats made more impulsive choices than 2 m.o.^29^. As a further confounder, 1 m.o. animals in a spatial (T-maze) DD task were shown to be more impulsive than 3 m.o. but only under very specific conditions (10 and 15 second delay)^30^. One major difference between DD and VDS paradigms is that, in the former, delay and reward-size effects cannot be isolated. When the amount of reward is controlled, and the indifference point calculated over an option between an adjusted delay and a variable (random) delay, adolescent animals are found to be less impulsive than young adult animals^51^, as also observed in the VDS – see also^34^. In this context, the relevant conclusion appears to be that reward-driven behavior in adolescents is more directed by an exogenous stimulus, while it is more goal-directed in adults^52^ – see also^53^. Indeed, adolescent and adult rats differ in their reward-evoked activities of the dorsal striatum and orbitofrontal cortex^54,55^ – see also for review^56^. Incongruence in the published results may also be partially attributed to differences in procedure, i.e. adjusting vs increasing delay^57^, ascending vs descending delay^58^ and magnitude of reinforcement^59^.

Female and male rats had similar rates of premature response in all blocks of the VDS paradigm, with exception of 3si. During this period females were seen to have a higher PR rate than males, potentially reflecting differences in action impulsivity previously observed in the training phase. Consistent with our overall findings, a number of earlier DD studies also found no significant sex-associated differences^39,60^. Other studies have reported small differences between male and female subjects under very particular experimental conditions^37^, while yet others have demonstrated an effect (females > males^36^), or even an opposite relationship (males > females^38^)^36^. In females, another variable which might contribute to the behavior under evaluation is phase of the estrous cycle. Our results show a trend towards increased tolerance to delay during diestrus compared to proestrus. Several studies in humans also have demonstrated diminished impulsivity during the mid phase of the menstrual cycle^61,62^. One interpretation of these differences is that they are related to the modulating effect by sex steroids upon dopaminergic tone^63–65^, which is known to affect impulsive behavior^4,5,66^.

An additional potential source of inter-study variability is the animals’ strain. It has for example been shown that male Lewis rats discount faster than Fisher 344^67–72^, although differences can be attenuated or eliminated with repeated assessment, perhaps as an effect of learning and/or aging^73^. Two other studies using additional rat strains^74,75^, support inter-strain differences. In our study, we restricted the analysis to the 12–18 m.o. group in order to evaluate groups comparable to one another. WH animals were found to have increased PR in comparison with SD rats, both during training and test, demonstrating strain differences in both action and delay intolerance components of impulsivity.

Finally, considering the heterogeneity of our population, differences in motivation and/or motor performance have necessarily to be accounted. Indeed, sex and age were associated with statistically significant differences in latency to feed, although these were in general small – maximum 244.6 ms (2–6 vs 12–18 m.o). Interestingly, younger (1–2 and 2–6 m.o.) and older (2–12 and 12–18 m.o.) groups differed substantially regarding response latencies (max. 2717.4 ms; 2–6 vs 12–18 m.o.). This is to some extent paradoxical in the sense that in the former animals need to move in a more elaborate manner (i.e. between the nosepoking and feeding orifices), while the latter requires minimal movement (the animal can perform sequential premature responses and eventually one final correct response). Such suggests that response latencies essentially reflect animals’ premature responding, i.e. animals responding at higher rates also have shorter latencies. In fact, females present both statistically significant higher PR and lower response latencies specifically at the 3si stage.

In conclusion, in this study of a sample of substantial size, we confirmed decreased action impulsivity and delay tolerance with age. Of interest was our finding that, in contrast to the prevailing view mainly derived from human studies^17–21,76^ – see also for review^22,23^, delay intolerance appears to be maximal at early adulthood, not in adolescence. In our analysis of action impulsivity, we found that females demonstrated a significantly greater number of premature responses than males. No similar difference was evident for delay tolerance. Our consideration of the influence of strain suggested that, in general, WH animals acted more impulsively than SD.

## Methods

### Subjects and experimental conditions

A total of 188 rats were used in this study (see below for details). Animals were kept in a room with controlled temperature (22 °C ± 1 °C), humidity (50–60%) and light cycle (12 hours; lights on at 8 a.m.) and were housed in groups of 2–3 in standard plastic cages with food and water available *ad libitum*. 2–3 days prior to the initiation of the VDS protocol food availability was restricted to 1 h per day. Animals’ weight was controlled throughout the protocol to prevent drops below 15% of baseline values. All procedures involving animals adhered to the guidelines of the European Communities Council Directive 2010/63/EU and were approved by the institutional ethics commission - Subcomissão de Ética para as Ciências da Vida e da Saúde (SECVS).

### Data collection

A database of all VDS records obtained in our institute was compiled. The data were obtained from 3 different sources: i. animals that performed the VDS task as controls for other experiments (mostly males), excluding any animal which had undergone any form of drug treatment or whose experimental records were incomplete; ii. animals that were specifically raised for this study (mostly females); and iii. aged animals which had primarily been used for breeding purposes prior to inclusion in this experiment. The final database contained a total of 188 entries. Groups were assembled by age: 1–2, 2–6, 6–12 and 12–18 months-old (m.o.), sex and strain: Sprague-Dawley (SD) and Wistar Han (WH) (Fig. 1A). Additionally, a group of young adult females were further classified according to the phase of their estrous cycle (see below).

### Variable delay-to-signal (VDS) task

The VDS protocol was administered as previously described^40^. Briefly reiterated, animals were tested 5-hole operant boxes (OB; 25 × 25 cm; TSE Systems, Germany) within a ventilated, sound attenuating environment. One of the OB walls contains five square apertures (#1-#5; 2.5 cm), elevated 2 cm from the grid floor. The opposing wall contains a similar aperture (#6) connected to a pellet dispenser. Each aperture contains a 3 W lamp bulb and an infrared beam which detects the activity of the animals.

#### Habituation

Animals were habituated to the OB in 2 daily sessions (a.m./p.m.; 4 hours apart) for 2 consecutive days (Fig. 1B). In the first 2 sessions (habituation day 1) animals were left to explore the OB for 15 minutes. For this phase, all lights were off and 10–15 sugar pellets (45 mg, Bioserv Inc., New Jersey,USA) were available at aperture #6 while apertures #1 to #5 were blocked with metallic caps. In sessions 3 and 4 (habituation day 2), animals were left to explore the OB for 30 minutes. Apertures #3 and #6 both were accessible and contained 3–5 and 10–15 pellets, respectively. During these sessions house light and aperture lights #3 and #6 were on.

#### Training

Training consisted of 2 daily sessions (a.m./p.m.; 4 hours apart) of 100 trials (or 30 minutes) each, for 5 consecutive days (Fig. 1B). These sessions were initiated by delivery of 1 pellet, after which the animals were trained to wait for 3 seconds (delay period). Aperture #3′s light was then turned on (response period) up to a maximum of 60 seconds. Nose pokes in the response period (correct responses) were rewarded with the delivery of a sugar pellet at aperture #6. Responses in the delay period (premature responses, PR) and omissions (absence of response) were punished with a timeout period (3 s) in complete darkness and no reward was delivered (Fig. 1C top). The house light was always on with exception of the timeout periods. In the training phase action impulsivity is measured in a manner akin to that of the 5-choice serial reaction time task^45,46^, i.e. by assessing the percentage of PRs.

#### Test

The VDS test consists of a single session of 120 trials (Fig. 1B). The test is similar to the training phase, except that nose pokes are allowed (i.e., not punished) (Fig. 1C bottom) and the delay periods are variable. The test starts with an initial block of 25 trials at 3 second delay (3si), followed by 70 trials of randomly distributed 6 or 12 second delays (6s and 12s) and concludes with a final block of 25 trials at 3 second delays (3sf). Two aspects of impulsive behavior can be evaluated by the VDS test. Action impulsivity is captured in the 1^st^ second of the delay and prematurity rate (PR rate) during the delays measures delay tolerance. The change in PR rate in the 3sf epoch after exposure to the longer intervals (6s and 12s) correlates with delay-discounting^40^.

PR rate is defined as the amount of PR per minute of total delay, PR/min

$${PR}/{\min }\,=\frac{{P}{{R}}_{{i}}}{{{N}}_{{i}}\times {{T}}_{{i}}}\times 60$$

where *PR*_*i*_ is the number of premature responses, *N*_*i*_ is the number of trials and *T*_*í*_ is the delay time for *i* = 3si, 6s, 12s or 3sf.

### Estrous cycle assessment

To determine the stage of the estrous cycle, the vaginal cytology method was used. The vaginal smear was performed after the VDS test. Cells from the smear were transferred to a dry glass slide and were air dried and stained with the Papanicolaou staining technique. Classification by stage (proestrus, estrus, metestrus and diestrus) was based on the presence or absence of nucleated epithelial cells, cornified epithelial cells and leukocytes, according to^77^; see also^78^.

### Statistical analysis

Statistical analyses were done using IBM SPSS Statistics 22 (IBM software, Inc., New York, USA). The analysis of training was performed by mixed-design ANOVA with session as the within-subjects’, and sex and age as between-subjects’ effects. The sphericity assumption was statistically assessed with Mauchly’s test. Comparisons between groups having one level were done applying one-way ANOVA and between groups with more than one level were done using two-way ANOVA. Bonferroni post-hoc correction was performed for multiple comparisons. Findings were considered significant at p < 0.05. All results are presented as mean ± SEM, unless otherwise clearly stated. Strain and estrous cycle comparisons were performed using sub-samples of the total database, as described in the results section.

## Electronic supplementary material

Supplementary Information

## Data Availability

The datasets generated during and/or analyzed during the current study are available from the corresponding author upon request.
