# Supplementary material for: Trait determinants of impulsive behavior: a comprehensive analysis of 188 rats
Source: Sci Rep. 2018 Dec 5;8:17666. doi: 10.1038/s41598-018-35537-7 (PMC6281674; doi:10.1038/s41598-018-35537-7)
Supplement: Supplementary file 1 — Supplementary Information [file 41598_2018_35537_MOESM1_ESM.docx]

**Trait determinants of impulsive behavior: a comprehensive analysis of 188 rats**

Ana Rosa Soares^1,2,‡^, Madalena Esteves^1,2,‡^, Pedro Silva Moreira^1,2^, Ana Margarida Cunha^1,2^, Marco Rafael Guimarães^1,2^, Miguel Murteira Carvalho^1,2,†^, Catarina Raposo-Lima^1,2^, Pedro Morgado^1,2^, Ana Franky Carvalho^1,2,3^, Bárbara Coimbra^1,2^, António Melo^1,2^, Ana João Rodrigues^1,2^, António José Salgado^1,2^, José Miguel Pêgo^1,2^, João José Cerqueira^1,2^, Patrício Costa^1,2^ Nuno Sousa^1,2^, Armando Almeida^1,2^, Hugo Leite-Almeida^1,2^*

1) Life and Health Sciences Research Institute (ICVS), School of Medicine, University of Minho, Braga, Portugal

2) ICVS/3B’s - PT Government Associate Laboratory, Braga/Guimarães, Portugal

3) Department of General Surgery, Hospital of Braga, Braga, Portugal

‡ equal contribution

† Present address: Kavli Institute for Systems Neuroscience and Centre for Neural Computation, Norwegian University of Science and Technology, Trondheim, Norway

** Corresponding author:* Hugo Leite-Almeida; School of Medicine, University of Minho; Campus de Gualtar; 4710-057 Braga, Portugal; Tel: +351-253-604931; E-mail: hugoalmeida@med.uminho.pt.


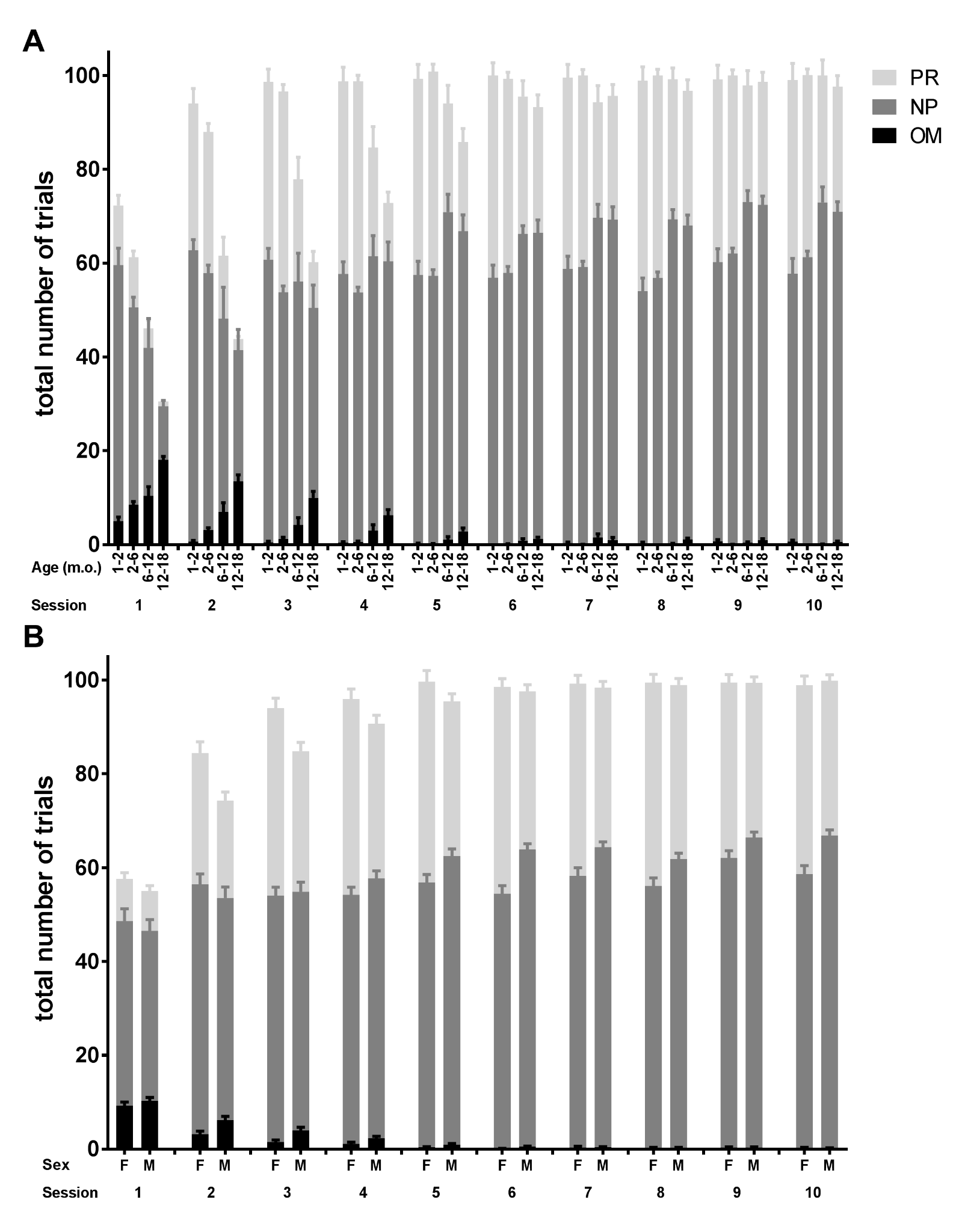


**Supplementary Fig. 1. Total number of trials during training.** Total number of trials as a sum of all omissions, correct nosepokes and premature responses according to age **(A)** and sex **(B)**. PR – premature responses; NP – correct nosepokes; OM – omissions; m.o. – months old; F – female; M – male.





**Supplementary Fig. 2. Plot of cumulative PRs in all blocks of the test.** PRs for each interval within each block - **(A)** 3si, **(B)** 6s, **(C)** 12s and **(D)** 3sf - are shown, grouped by age (left) and sex (right). The x axis shows intervals (in miliseconds) within each block. PR - premature response; m.o - months old.





**Supplementary Fig. 3. Influence of estrous cycle upon choice and delay intolerance impulsivity.** The analysis of the effect of the estrous cycle in impulsive behavior was restricted to 2-6 m.o. females in the diestrus or proestrus phase of the cycle. The phase of the estrous cycle did not affect **(A)** impulsive actions in the first second of the 3si block of the test, **(B)** PR rate in any block or **(C)** PR rate 3sf/3si comparison. Data is presented as mean ± SEM. PR rate - rate of premature responses per minute.

|  | | | %OM Effects | | | | |
| --- | --- | --- | --- | --- | --- | --- | --- |
|  |  |  | session/age | | | | session/sex |
|  |  |  | 1-2 | 2-6 | 6-12 | 12-18 |  |
| %PR Effects | session/age | 1-2 |  | <0.010 | <0.001 | <0.001 | 0.376 |
|  |  | 2-6 | 0.469 |  | <0.010 | <0.001 | 0.418 |
|  |  | 6-12 | 0.396 | <0.050 |  | <0.050 | <0.010 |
|  |  | 12-18 | <0.010 | <0.001 | 0.109 |  | <0.010 |
|  | session*sex | | 0.058 | 0.110 | 0.126 | <0.001 |  |

**Supplementary Table 1. P-values of effects found in the comparison of the different age groups on learning and action impulsivity.** Effects on task learning were assessed through the percentage of omissions (%OM - white area) while effects on action impulsivity were evaluated in the percentage of premature responses (%PR - grey area) during training. Main session/group (within/between) effects are shown for the comparison between two age groups and comparison between males and females within the same age group. P<0.05 was considered the threshold for statistical significance and age is measured in months.

|  | | | age | | | |
| --- | --- | --- | --- | --- | --- | --- |
|  |  |  | 1-2 | 2-6 | 6-12 | 12-18 |
|  |  |  | 3si | | | |
| age | 1-2 | 6s |  | 1.000 | 0.698 | <0.010 |
|  | 2-6 |  | 0.146 |  | 0.799 | <0.001 |
|  | 6-12 |  | 1.000 | 1.000 |  | 0.619 |
|  | 12-18 |  | <0.050 | <0.001 | <0.010 |  |
|  | | | 12s | | | |
| age | 1-2 | 3sf |  | 0.148 | 1.000 | <0.050 |
|  | 2-6 |  | <0.010 |  | 1.000 | <0.001 |
|  | 6-12 |  | 1.000 | <0.010 |  | <0.010 |
|  | 12-18 |  | 0.115 | <0.001 | 0.808 |  |
|  | | | log(3sf/3si) | | | |
| age | 1-2 | |  | 0.070 | 1.000 | 0.582 |
|  | 2-6 | |  |  | 0.129 | <0.001 |
|  | 6-12 | |  |  |  | 1.000 |
|  | 12-18 | |  |  |  |  |

**Supplementary Table 2. P-values of effects found in the comparison of the different age groups on delay intolerance.** Effects were assessed based on the prematurity rate (PR rate) during the test phases (3si - top white area, 6s - top grey area, 12s - middle white area and 3sf - middle grey area) and in the 3sf normalized to baseline (log(3sf/3si) - bottom white area). Main effects of group (age interval), are shown. P<0.05 was considered the threshold for statistical significance and age is measured in months.

|  | Test | | | | | |
| --- | --- | --- | --- | --- | --- | --- |
|  | PR rate 1^st^ sec effects | PR rate effects | | | | |
|  |  | 3si | 6s | 12s | 3sf | log(3sf/3si) |
| Estrous Cycle | F_(1,14)_=0.751; p=0.402 | F_(1,12)_=0.93; p=0.355 | F_(1,13)_=1.92; p=0.189 | F_(1,12)_=3.50; p=0.086 | F_(1,12)_=1.14; p=0.307 | F_(1,14)_<0.01; p=0.997 |

**Supplementary Table 3. Effect of estrous cycle phase on action impulsivity and delay intolerance.** Effects were assessed based on the prematurity rate (PR rate) during the first second of the test, during the several test phases (3si, 6s, 12s and 3sf) and in the 3sf normalized to baseline (log(3sf/3si)). Main effects of group (estrous cycle) is shown. P<0.05 was considered the threshold for statistical significance.
